# Targeted siRNA Delivery Against RUNX1 Via tFNA: Inhibiting Retinal Neovascularization and Restoring Vessels Through Dll4/Notch1 Signaling

**DOI:** 10.1167/iovs.66.3.39

**Published:** 2025-03-19

**Authors:** Xiaodi Zhou, Xiaoxiao Xu, Qiong Wang, Yanting Lai, Linyan Zhang, Yunfeng Lin, Xiaoyan Ding, Limei Sun

**Affiliations:** 1 State Key Laboratory of Ophthalmology, Zhongshan Ophthalmic Center, Sun Yat-Sen University, Guangdong Provincial Key Laboratory of Ophthalmology and Visual Science, Guangzhou, China; 2 Innovative Institute of Chinese Medicine and Pharmacy, Chengdu University of Traditional Chinese Medicine, Chengdu, China; 3 State Key Laboratory of Oral Diseases, West China Hospital of Stomatology, Department of Maxillofacial Surgery, West China Stomatological Hospital, Sichuan University, Chengdu, China

**Keywords:** tetrahedral framework nucleic acids, small interfering RNA, RUNX1, retinal neovascularization, vessel supply restoring

## Abstract

**Purpose:**

To assess the efficacy of tetrahedral framework nucleic acids (tFNAs) as a delivery system for small interfering RNA (siRNA) targeting RUNX1 (siRUNX1) in inhibiting retinal neovascularization (RNV) and restoring vascular integrity via the Dll4/Notch1 signaling pathway.

**Methods:**

tFNAs and tFNAs-siRUNX1 were synthesized using annealing of single-stranded DNAs and characterized by PAGE and high-performance capillary electrophoresis. Human umbilical vein endothelial cells were treated under hypoxic conditions with tFNAs-siRUNX1, and cellular uptake was evaluated using fluorescence microscopy and flow cytometry. Angiogenesis was assessed through EdU proliferation, tube formation, and wound-healing assays. In vivo experiments used oxygen-induced retinopathy (OIR) and laser-induced choroidal neovascularization (CNV) models in mice, with subsequent imaging by optical coherence tomography (OCT) and fundus fluorescence angiography. Gene and protein expression were analyzed by RT-PCR and Western blotting, focusing on the Dll4/Notch1 pathway and apoptosis markers.

**Results:**

tFNAs-siRUNX1 effectively inhibited endothelial cell proliferation, migration, and tube formation in vitro. In OIR and CNV models, it reduced neovascularization, nonperfusion areas, and vascular leakage. The mechanism involved modulation of the Dll4/Notch1 pathway, with decreased Dll4, Notch1, and Hes1 and increased Nts expression. tFNAs-siRUNX1 also reduced endothelial cell apoptosis via the Bcl-2/Bax pathway.

**Conclusions:**

tFNAs-siRUNX1 is a promising delivery system for targeting RNV, inhibiting neovascularization, and restoring retinal vascular integrity, providing a potential therapeutic alternative to anti-VEGF treatments.

Retinal neovascularization (RNV) is a primary cause of vision loss and blindness around the world, characterized by the formation of abnormal new vessels in the retina. These vessels lack the normal vascular structure and can leak fluid, rupture, bleed, or form scar tissue, ultimately leading to vision loss. RNV represents a complex vascular anomaly underlying various vision-threatening retinopathies across different age groups, such as retinopathy of prematurity,^1^ proliferative diabetic retinopathy,^2^^,^^3^ and wet age-related macular degeneration.^4^ The primary mechanism involves retinal hypoxia due to insufficient vascular supply, which could result from poor vessel development, capillary loss, and vascular obstruction, leading to ischemia and subsequent abnormal vessel growth.^5^ Currently, the first-line treatment for RNV is anti-VEGF therapy, but it has notable limitations. This includes complications like vitreous hemorrhage, vitreoretinal traction, neuronal toxicity, and vascular occlusions. Most importantly, this therapy does not address retinal ischemia or contribute to retinal revascularization.^6^ This highlights the urgent need for new targets for these diseases that not only inhibit abnormal vessel growth but also address ischemia and restore the retinal blood supply.^7^ Discovering such groundbreaking treatments is crucial for effectively managing and ultimately curing these diseases.^5^

Recent research has spotlighted the runt-related transcription factor 1 (RUNX1) as a promising target for RNV treatment, given its significant role in ischemic retinopathy.^8^^–^^10^ RUNX1 serves as a key biomarker for RNV, making it a promising target for correcting abnormal vascular growth and promoting retinal revascularization. However, current approaches face limitations: Ro5-3555, an indirect RUNX1 inhibitor, is confined to laboratory use due to poor solubility, large molecular weight, and uncertain human safety, Similarly, small interfering RNA (siRNA) targeting RUNX1 (siRUNX1) can reduce neovascularization but suffers from degradation, cell entry issues, and immunogenicity.^11^^,^^12^ Therefore, there is an urgent need for a safe and efficient drug delivery system.

Building on this, our previous research has highlighted tetrahedral framework nucleic acids (tFNAs) as a promising delivery system in biology due to their exceptional properties.^13^^,^^14^ Studies have shed light on the utility of tFNAs in managing ocular conditions. For instance, tFNAs have successfully delivered microRNA-22-3p (miR-22) to impaired retinal neurons, providing neuroprotection in glaucoma.^14^^,^^15^ Herein, we develop a versatile tFNA system, specifically designed to deliver siRUNX1, termed tFNAs-siRUNX1. Our research reveals that tFNAs efficiently and safely delivered siRUNX1 to target endothelial cells in hypoxic and ischemic retinas, significantly reducing neovascularization, alleviating retinal ischemia, and restoring blood supply, demonstrating tFNAs-siRUNX1’s potential in addressing retinal neovascularization disorders. Notably, this approach enhances neurotensin (Nts) activation, prevents endothelial cell apoptosis, and modulates the Dll4/Notch1 signaling pathway. This strategy holds great promise as a crucial agent for RNV and could potentially provide a definitive solution for curing RNV, with significant implications for future clinical applications.

## Material and Methods

### Synthesis of tFNAs and tFNAs-siRUNX1

Four single-stranded DNAs (ssDNAs) were synthesized and characterized by Genescript (Nanjing, China). First, a TM buffer with a pH of 8.0, containing 10 mM Tris-HCl and 50 mM MgCl_2_, was prepared. Equal concentrations of the four ssDNAs were poured into TM buffer. After thorough mixing and centrifugation, the mixed solution was heated to 95°C for 10 minutes and then cooled to 4°C for 20 minutes to produce tFNAs. For the preparation of tFNAs-siRUNX1, three ssDNAs (ssDNA-S2, S3, S4) and a ssDNA modified by siRNA against *RUNX1* (siRUNX1) were synthesized according to the same method as tFNAs. The sequences of the four ssDNAs and siRUNX1 are shown in Table 1. Then, 100 µL of the tFNAs-siRUNX1 sample was retained after synthesis as a prepurification sample and introduced into a high-performance liquid chromatography (HPLC) system. tFNAs-siRUNX1 were then purified with a DNA Pac PA100 (ThermoFisher Scientific, Massachusetts, USA) chromatographic column at a flow rate of 10 mL·min^−1^ using different mobile phases (mobile phase A: 25 mM Tris-HCL, mobile phase B: 25 mM Tris-HCL + 375 mM NaClO_4_ C. 25 mM Tris). Next, the main peak of the sample was collected, and purity was evaluated using HPLC.

**Table 1.** The Sequences of the Four ssDNAs and siRNA Against *RUNX1*

ssDNA	Base Sequence (5′-3′)
S1	ATTTATCACCCGCCATAGTAGACGTATCACCA
	GGCAGTTGAGACGAACATTCCTAAGTCTGAA
S2-Cy5	ACATGCGAGGGTCCAATACCGACGATTACAG
	CTTGCTACACGATTCAGACTTAGGAATGTTCG
S3	ACTACTATGGCGGGTGATAAAACGTGTAGCAA
	GCTGTAATCGACGGGAAGAGCATGCCCATCC
S4	ACGGTATTGGACCCTCGCATGACTCAACTGCC
	TGGTGATACGAGGATGGGCATGCTCTTCCCG
siRNA against *RUNX1* (sense)	GGCAGAAACUAGAUGAUCAGA
siRNA against *RUNX1* (antisense)	UGAUCAUCUAGUUUCUGCCGA

### Characterization of tFNAs and tFNAs-siRUNX1

We conducted experiments, following methods described in previous studies, to verify the successful synthesis of tFNAs.^1^^,^^2^ Briefly, we employed PAGE and high-performance capillary electrophoresis (HPCE) to ascertain the molecular weight differences between tFNAs and tFNAs-siRUNX1. Additionally, we used a nanoparticle size analyzer to detect variations in zeta potential and particle sizes of the two compounds, determining the success of the synthesis.

### Cell Culture and Treatment

Human umbilical vein endothelial cells (HUVECs) were acquired from ATCC (Manassas, VA, USA). We incubated the cells with 1× Dulbecco's Modified Eagle's Medium Nutrient Mixture F-12 (DMEM/F-12 basic; Gibco, Massachusetts, USA) supplemented with 10% fetal bovine serum (FBS; Gibco) and 100% antibiotic solution (10,000 U·mL^−1^ penicillin and 10,000 µg·mL^−1^ streptomycin; Gibco) under normoxia (95% air and 5% CO_2_ at 37°C) for 24 hours. Next, HUVECs were cultured under normoxia (37°C, 5% CO_2_) or hypoxia (37°C, 1% O_2_, 5% CO_2_) with various concentrations (vehicle [VHCL], 10 µg·µL^−1^ aflibercept [AFL], 1 nM siRUNX1 with Lipofectamine RNAiMAX Transfection Reagent [ThermoFisher Scientific], 100 nM tFNAs, and 62.5 nM, 100 nM, or 250 nM tFNAs-siRUNX1) and the original DMEM/F-12 containing 10% FBS accordingly for another 24 hours.

### Uptake of Cy5-Loaded tFNAs-siRUNX1

Previous investigations have indicated successful uptake of tFNAs into RAW264.7 cells and HUVECs.^1^^,^^3^ In this experiment, we examined the uptake of tFNAs-siRUNX1 into HUVECs. One ssDNAs (S2) was labeled with Cy5 fluorescence. HUVECs were cultured in 6-well plates (Corning, New York, NY, USA) at a density of 3 × 10^5^ cells per well. After the cells were cultured for 24 hours, Cy5-tFNAs-siRUNX1 was added for another 24-hour incubation, and then we measured the fluorescence intensity at 24 hours and compared it with the blank control group and siRUNX1 without transfection reagent. Samples meeting the requirements of flow cytometry were collected. Flow cytometry (Attune NxT; ThermoFisher Scientific) was used to spot the intracellular fluorescence intensity at 24 hours.

### Cell Immunofluorescence Assay

Cell immunofluorescence staining was conducted to evaluate the expression of proteins related to angiogenesis and cell apoptosis. HUVECs were plated in 6-well plates at a density of 9.6 × 10^5^ cells and treated as previously mentioned. Then, the cells were fixed with 4% paraformaldehyde (PFA) in PBS for 15 minutes and permeabilized and blocked with 0.5% Triton X-100 (Biofroxx, Einhausen, Germany) and 1% BSA (Biosharp, Anhui, China) for 20 minutes. Then, the samples were incubated together with the following: diluted Dll4 (1:100, ER1706-29; HUABIO, Hangzhou, China), Notch1 (1:100, ER1606-55; HUABIO), Hes1 (1:100, ab108937; Abcam, Cambridge, MA, USA), Nts (1:100, ER65615; HUABIO), Bcl-2 (1:100, ab59348; Abcam), and Bax (1:100, ab216494; Abcam) primary antibody at 4°C overnight. Next, cells were cultured with the secondary antibody anti-rabbit IgG (1:1000, 4413; Cell Signaling Technology [CST], Massachusetts, USA) under ambient temperature for 2 hours. Then, the cell nucleus was stained with Hoechst 33342 (Solarbio, Beijing, China), and the cytoskeleton was stained with phalloidin (Solarbio). Once stained, the samples were preserved using anti-fluorescence-quenching sealing tablets.

### Real-Time Fluorescence Quantitative PCR

We employed real-time fluorescence quantitative PCR (RT-PCR) technology to detect the expression of related genes. Total RNA was extracted with TRNzol Universal (TIANGEN, Beijing, China). Following extraction, we purified and reverse-transcribed the RNA using the FastKing RT Kit With gDNase (TIANGEN). All target mRNAs were amplified by quantitative RT-PCR using the SYBR Green Realtime PCR Master Mix (TOYOBO, Shanghai, China). Table 2 provides the sequences of the primers for the genes under investigation, namely *Dll4*, *Nocth1*, *Hes1*, *Nts*, *Bcl-2* and *Bax*. All these primers were designed based on a BLAST search, with β-actin serving as the amplification control.

**Table 2.** The Sequences of PCR Primers

Gene	Primer Sequence (5′-3′)
*Dll4*	Forward: TGGGTCAGAACTGGTTATTGGA
	Reverse: GTCATTGCGCTTCTTGCACAG
*Notch1*	Forward: GAGGCGTGGCAGACTATGC
	Reverse: CTTGTACTCCGTCAGCGTGA
*Hes1*	Forward: TGTCAACACGACACCGGATAA
	Reverse: AATGCCGCGAGCTATCTTTCT
*Nts*	Forward: GGGCTTTTCAACACTGGGAGTTAAT
	Reverse: AGCTGCCGTTTCAGAATATAAGG
*Bcl-2*	Forward: TGAACCGGCATCTGCACAC
	Reverse: CGTCTTCAGAGACAGCCAGGAG
*Bax*	Forward: CGGCGAATTGGAGATGAACTG
	Reverse: AGCAAAGTAGAAGAGGGCAACC

### Protein Extraction and Western Blot Analysis

HUVECs were cultured, categorized into distinct groups, and subsequently treated as described. After 24 hours, proteins were extracted from the HUVECs using RIPA (Solarbio) containing protease inhibitors. The protein concentrations were determined by the BCA Protein Quantitation Assay Kit (KeyGEN, Nanjing, China) using protein standard solution as a standard. Samples of supernatants containing 20 µg of protein were heated to 95°C for 5 minutes and separated with SurePAGE, Bis-Tris, 10 × 8, 4–20%, 12 wells (GenScript, New Jersey, USA) and subsequently transferred onto a polyvinylidene difluoride filter (PVDF) membrane (Millipore, Merck, Germany). After blocking with 5% defatted milk in TBS-Tween-20 (TBST) for 1 hour at room temperature, the PVDF membranes were incubated with diluted Dll4 (1:1000, ER1706-29; HUABIO), Notch1 (1:1000, ER1606-55; HUABIO), Hes1 (1:1000, ab108937; Abcam), Nts (1:1000, ER65615; HUABIO), Bcl-2 (1:1000, ab59348; Abcam) and Bax (1:1000, ab216494; Abcam), and β-actin (1:1000, 4967S; CST) antibodies in blocking solution overnight at 4°C. The following day, membranes underwent a series of five washes. Subsequently, they were incubated with the anti-rabbit IgG, HRP-linked Antibody (1:3000, 7074; CST) at room temperature for 2 hours. Visualization was achieved using an enhanced chemiluminescence system (ProteinSimple, California, USA).

### Angiogenesis Experiments

We processed the HUVECs in groups (VHCL, 10 µg·µL^−1^ AFL, 1 nM siRUNX1, 100 nM tFNAs, or 100 nM tFNAs-siRUNX1) using the aforementioned methods.

#### EdU Cell Proliferation Assay

To confirm that cell proliferation was inhibited by tFNAs-siRUNX1, EdU was detected using Alexa Fluor 488 Click-iT EdU Imaging Kits (ThermoFisher Scientific). HUVECs were seeded in 6-well plates and cultured for 24 hours, following by treatments described above. The EdU cell proliferation assay was executed in alignment with the manufacturer's guidelines. Fluorescence staining images of the HUVECs in 6-well plates were captured using a confocal laser microscope (Carl Zeiss, Oberkochen, Germany). ImageJ software (National Institutes of Health, Bethesda, MD, USA) facilitated the statistical analysis. Each experiment was conducted at least three times.

#### Tube Formation Assay

A tube formation experiment was performed to explore the influence of tFNAs-siRUNX1 on HUVECs angiogenesis. HUVECs were seeded in 6-well plates and cultured for 24 hours. Matrigel solution (50 µL per well) was added to a 96-well plate (kept on ice) and incubated at 37°C for 30 minutes to allow gel formation. Then, 50 µL HUVECs (approximately 1.0 × 10^5^ cells·mL^−1^) with VHCL, 37.50 µL HUVECs (approximately 1.0 × 10^5^ cells·mL^−1^) with 12.5 µL of 40 µg·µL^−1^ AFL (1 µg·µL^−1^), 50 µL HUVECs (approximately 1.0 × 10^5^ cells·mL^−1^) transfected with 1 nM siRUNX1, 45 µL HUVECs (approximately 1.0 × 10^5^ cells·mL^−1^) with 5 µL of 1 µM tFNAs (100 nM), and 45 µL HUVECs (approximately 1.0 × 10^5^ cells·mL^−1^) with 5 µL of 1 µM tFNAs-siRUNX1 (100 nM) were seeded in the prepared 96-well plates (containing gels) after incubation under normoxia (37°C, 5% CO_2_) or hypoxia (37°C, 1% O_2_, 5% CO_2_) with the original DMEM/F-12 containing 10% FBS accordingly. Twelve hours later, three different wells per group were photographed using the Inverted Biologic Microscope (IX2-SL; Olympus Corporation, Tokyo, Japan). Then tube formation on images was quantified using the Angiogenesis Analyzer plugin for ImageJ. Experiments were performed a minimum of three times.

#### Cell Migration Assay

To explore the influence of tFNAs-siRUNX1 on HUVEC migration, we conducted a wound-healing experiment. HUVECs were seeded in 6-well plates and cultured for 24 hours. The cells were divided into six groups, and appropriate treatments (VHCL, 10 µg·µL^−1^ AFL, 1 nM siRUNX1, 100 nM tFNAs, or 100 nM tFNAs-siRUNX1) were added to the culture medium (without growth factors or FBS). Then, a scratch was made with a sterile pipette tip, and wound images were taken after incubation under normoxia (37°C, 5% CO_2_) or hypoxia (37°C, 1% O_2_, 5% CO_2_) for 0, 24, and 48 hours under the Inverted Biologic Microscope (IX2-SL; Olympus Corporation). ImageJ software was used for statistical analysis. Experiments were performed a minimum of three times.

### Laser-Induced Choroidal Neovascularization Model

Male C57BL/6J mice, aged 6 to 8 weeks, were sourced from GemPharmatech Co., Ltd (Nanjing, China). To prepare for the procedure, their pupils were first dilated using compound tropicamide. Subsequently, the mice were anesthetized with a 1% pentobarbital sodium solution at a dosage of 50 mg·kg^−1^. Using a laser photocoagulation apparatus (Supra 810; Quantel Medical, Paris, France), laser photocoagulation was administered. The specific parameters included a laser spot diameter of 50 µm, a duration of 100 ms, a 280-mW intensity, and four spots per eye. Care was taken to ensure that the laser spots were positioned 2.5 to 3-disc diameters away from the optic nerve head, avoiding major blood vessels. The formation of a white bubble at each laser spot signified a rupture in Bruch's membrane, which typically precedes choroidal neovascularization (CNV) development.

### Oxygen-Induced Retinopathy Model

C57BL/6J mice were acquired from GemPharmatech Co., Ltd. Oxygen-induced retinopathy (OIR) was carried out in C57BL/6J mice as previously described by our previous study.^1^ All animal studies were approved by the Institutional Animal Care and Use Committee of Zhongshan Ophthalmic Center.

### Intravitreal Injection

First, mice were anesthetized with 1% pentobarbital sodium (50 mg·kg^−1^). Next, 1 µL AFL (40 µg·µL^−1^), siRUNX1 (10 nM) without transfection reagent, tFNAs (1 µM), or tFNAs-siRUNX1 (1 µM) was injected into the vitreous cavity. The injections were carefully administered using a 33-gauge Hamilton syringe (Hamilton, Reno, USA) under a stereomicroscope (M620 F20; Leica Microsystems, Wetzlar, Germany) to sidestep any potential damage to the lens. These injections took place at postnatal 7 (P7) or postnatal 12 (P12) in OIR mice. For the control, 1 µL of a vehicle solution was injected intravitreally. For CNV mice, 4 days after laser treatment, 1 µL tFNAs-siRUNX1 (1 µM) was injected into the vitreous cavity. As a control, an equivalent volume of vehicle/AFL/siRUNX1/tFNAs was injected using a 31-gauge Hamilton syringe (Hamilton). Postoperation, a tobramycin eye ointment was applied as a preventative measure against potential infections.

### Optical Coherence Tomography (OCT) and Fundus Fluorescence Angiography (FFA)

On day 4 (D4) and day 7 (D7) after laser, leakage and size of neovascularization were assessed in the mice using fundus fluorescence angiography (FFA) and optical coherence tomography (OCT). The instrument was a retinal imaging microscope (Phoenix Micron IV, Phoenix, AZ, USA). Briefly, mice were anesthetized with 1% pentobarbital sodium (50 mg·kg^−1^) after dilating the pupils. OCT images were captured when CNV lesions were most clearly imaged. FFA images were captured at appropriate intervals after an intraperitoneal injection of 0.2 mL of 2% sodium fluorescein. The OCT and FFA images captured at the same time point were analyzed by ImageJ software. Leakage areas and intensity were measured.

### Whole-Mounted Retinal Immunofluorescence

Mice were euthanized at P8 or P17, and eyes were enucleated and fixed with freshly prepared 4% PFA for 1 hour. Using a stereo operating microscope (MZ62; Mshot, Guangdong, China), the retinas were carefully dissected. These intact retinas were then blocked and permeabilized using a solution of PBS with 1% BSA and 0.5% Triton X-100. Afterward, the retinas were incubated with the primary antibody, IB4 conjugated to Alexa Fluor 488 (1:200, I21411; ThermoFisher), anti–ETS-related gene (ERG) antibody (1:600, 97249; CST), and anti–collagen IV antibody (1:600, ab19808; Abcam) overnight at 4°C. Following this, the retinas were washed in PBS, mounted onto slides, and examined using an inverted microscope (Ts2FL; Nikon, Tokyo, Japan).

### Measurement of Retinal Neovascularization

To determine the extent of RNV, retinal images from various treatments were randomized, labeled, and analyzed, focusing on RNV clock hours and the RNV area relative to the total retinal area. Two masked reviewers performed all analyses. The presence of RNV was determined with a technique adapted from those used in clinical trials and animal model determination. For the clock hour assessment, retinal flat mounts were divided into 12 clock hours of approximately equal area using Adobe Photoshop (Adobe, Sa Jose, CA, USA) and assessed for the presence of RNV.^4^ RNV area and total retinal area were quantified using ImageJ software. The area within each clock hour showing RNV was measured, summed to give the total RNV area for each retina, and then represented as a percentage of the full retinal area.

### Statistical Analysis

Statistical analyses were performed using SPSS Statistics 25.0 (SPSS, Inc., Chicago, IL, USA). Data from multiple repeated experiments are presented as mean ± standard deviation (SD). To ascertain significant differences between groups, a one-way analysis of variance was employed, with *P* values below 0.05 indicating statistical significance. The number of repetitions and/or total animals involved are detailed in the figure legends or directly on the figures.

## Results

### Synthesis and Characterization of tFNAs-siRUNX1

**Figure 1.**
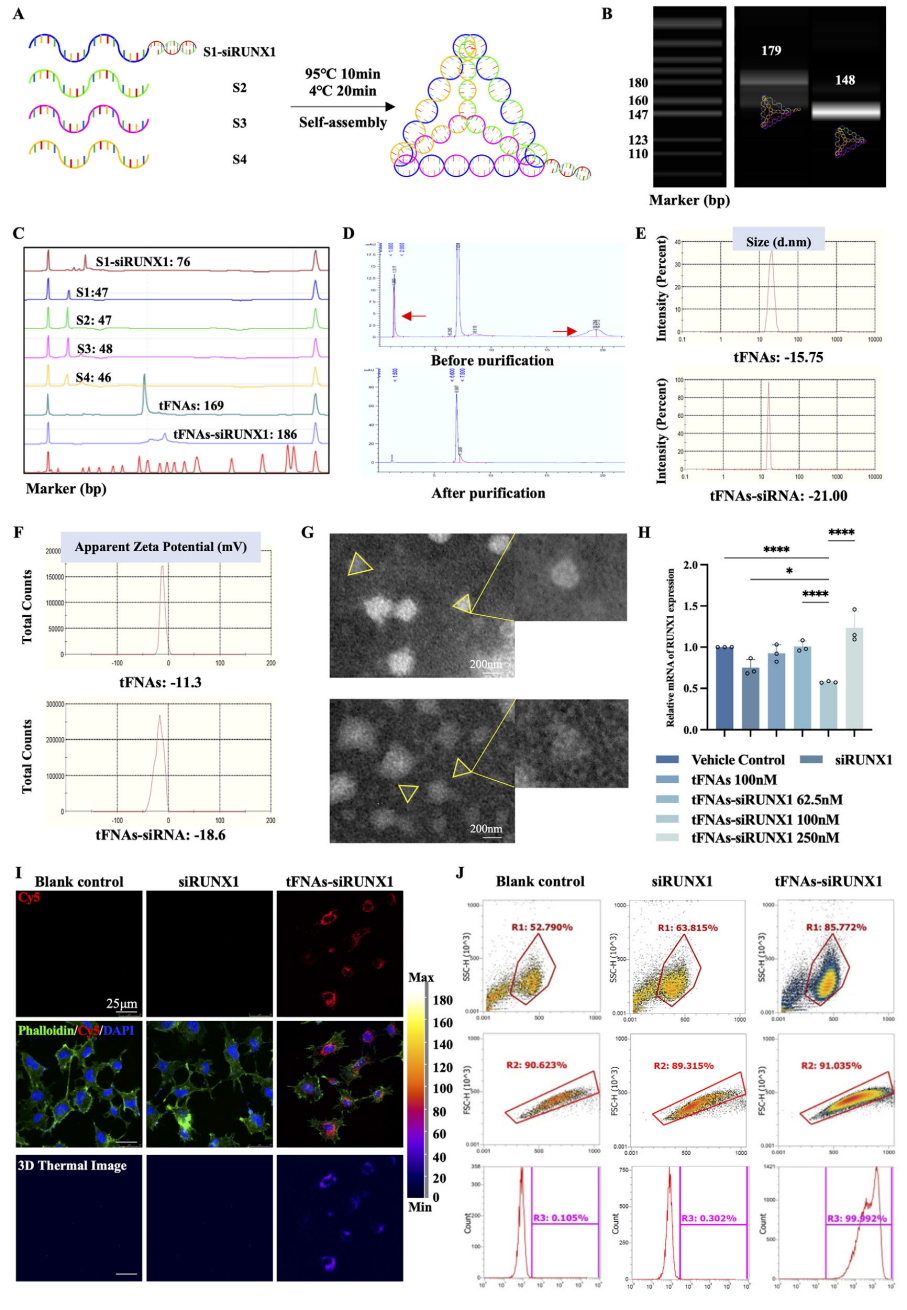
Synthesis and characterization of tFNAs-siRUNX1. (**A**) Diagram of the synthesis process. (**B**) PAGE showing molecular weights of tFNAs and tFNAs-siRUNX1. (**C**) HPCE confirming successful generation of tFNAs and tFNAs-siRUNX1. (**D**) HPLC results before and after purification, highlighting removal of inconsistent bases or strands. (**E**) Dynamic light scattering analysis characterizing tFNAs and tFNAs-siRUNX1. (**F**) Zeta potential analysis indicating stability. (**G**) Transmission electron microscopy image showing molecular structure. *Scale bars*: 200 nm. (**H**) RT-PCR analysis of RUNX1 expression under hypoxia. (**I**) Uptake of tFNAs-siRUNX1 by endothelial cells after 8 hours. *Scale bars*: 25 µm. (**J**) Flow cytometry showing higher uptake of siRUNX1 in tFNAs-siRUNX1 (99.9%) compared to siRUNX1 alone (0.302%) and control (0.105%). R1 (*top*) represents the SSC-H, which corresponds to the main cell population in the sample. R2 (*middle*) represents cells after clump removal, and R3 (*bottom*) indicates the positive labeling rate.

The documented literature emphasized the convenience of assembling tFNAs using a straightforward annealing process with four specifically designed ssDNAs (Table 1).^16^^–^^18^ In our study, we modified a siRUNX1 by attaching it to the 3′ end of S1 ssDNA, creating a new molecule, termed S1-siRUNX1. This attachment was facilitated by a -TTTTT- linker sequence, binding the sense strand of siRUNX1 to S1. We then synthesized tFNAs-siRUNX1 by incorporating S2, S3, and S4 together (Fig. 1A).

The detection of tFNAs and tFNAs-siRUNX1 was achieved through 8% PAGE (Fig. 1B). The results revealed that tFNAs-siRUNX1 migrated more slowly than tFNAs, indicating a higher molecular mass contributed to the successful attachment of siRUNX1 to tFNAs. We then employed HPCE to validate and purify the nanomaterials (Fig. 1C). HPCE showed a significant increase in the purity of tFNAs-siRUNX1, with impurities indicated by red arrows in Fig. 1D.

Next, further characterization of tFNAs and tFNAs-siRUNX1 was undertaken using dynamic light scattering, apparent zeta potential, and transmission electron microscopy, respectively (Figs. 1E–G). The results indicated that tFNAs had a triangular structure with an average particle size of 15.75 nm. The zeta potential of tFNAs was about −11.3 mV, attributed to the inherent negative charge of nucleic acid molecules.^14^^,^^19^ tFNAs-siRUNX1, while also exhibiting a tetrahedral structure akin to a triangle, had a larger particle size, estimated at 21.00 nm, with the zeta potential of −18.6 mV. The location of siRUNX1 was discerned at the vertex of this tetrahedral structure. Taken together, these findings robustly indicate the successful incorporation of siRUNX1 into the tFNA framework.

To determine the optimal working concentration, we observed that RUNX1 expression decreased more noticeably after treating with 100 nM tFNAs-siRUNX1 under hypoxic conditions (37°C, 1% O_2_, 5% CO_2_), if compared to treatments with 100 nM tFNAs, 62.5 nM tFNAs-siRUNX1, or 250 nM tFNAs-siRUNX1 (Fig. 1H). Interestingly, among the three concentrations (62.5 nM, 100 nM, and 250 nM), the middle concentration, 100 nM, showed the most significant inhibition of RUNX1 expression, which does not appear to follow a typical dose–response relationship. We hypothesize that this could be due to several factors, such as the saturation of the RNA silencing mechanism at a certain concentration or potential off-target effects at higher concentrations that might reduce the efficacy of RUNX1 inhibition. Another possibility is that the cellular uptake or processing of tFNAs-siRUNX1 may be optimized at 100 nM, leading to the most effective knockdown of RUNX1 at this concentration. Further investigations are needed to fully elucidate the underlying mechanisms. Furthermore, this concentration was much lower than observed in our previous studies,^17^ which may be attributed to the improved purification process from removing excess and mismatched bases along with stray single strands. We also confirmed that tFNAs-siRUNX1 can penetrate HUVECs, which are commonly used in studies of retinal vascular diseases due to their similarity to retinal microvascular endothelial cells. HUVECs were incubated with Cy5-labeled siRUNX1 and tFNAs-siRUNX1, respectively, for 24 hours, and the Cy5 localization was assessed using immunofluorescence assays. tFNAs-siRUNX1 predominantly settled in the cytoplasm (Fig. 1I). To verify the efficiency of tFNAs in carrying siRUNX1, flow cytometry was used to measure the uptake of siRUNX1 and tFNAs-siRUNX1 quantitatively. The results indicated that the cellular uptake efficiency of tFNAs-siRUNX1 was remarkably higher (99.992%) than the blank control (0.105%) and siRUNX1 groups (0.302%), respectively (*P <* 0.0001, Fig. 1J). Thus, the concentration 100 nM was adopted for all subsequent in vitro and in vivo experiments.

### tFNAs-siRUNX1 Inhibits the Proliferation, Tube Formation, and Migration of Endothelial Cells Under Hypoxia In Vitro

**Figure 2.**
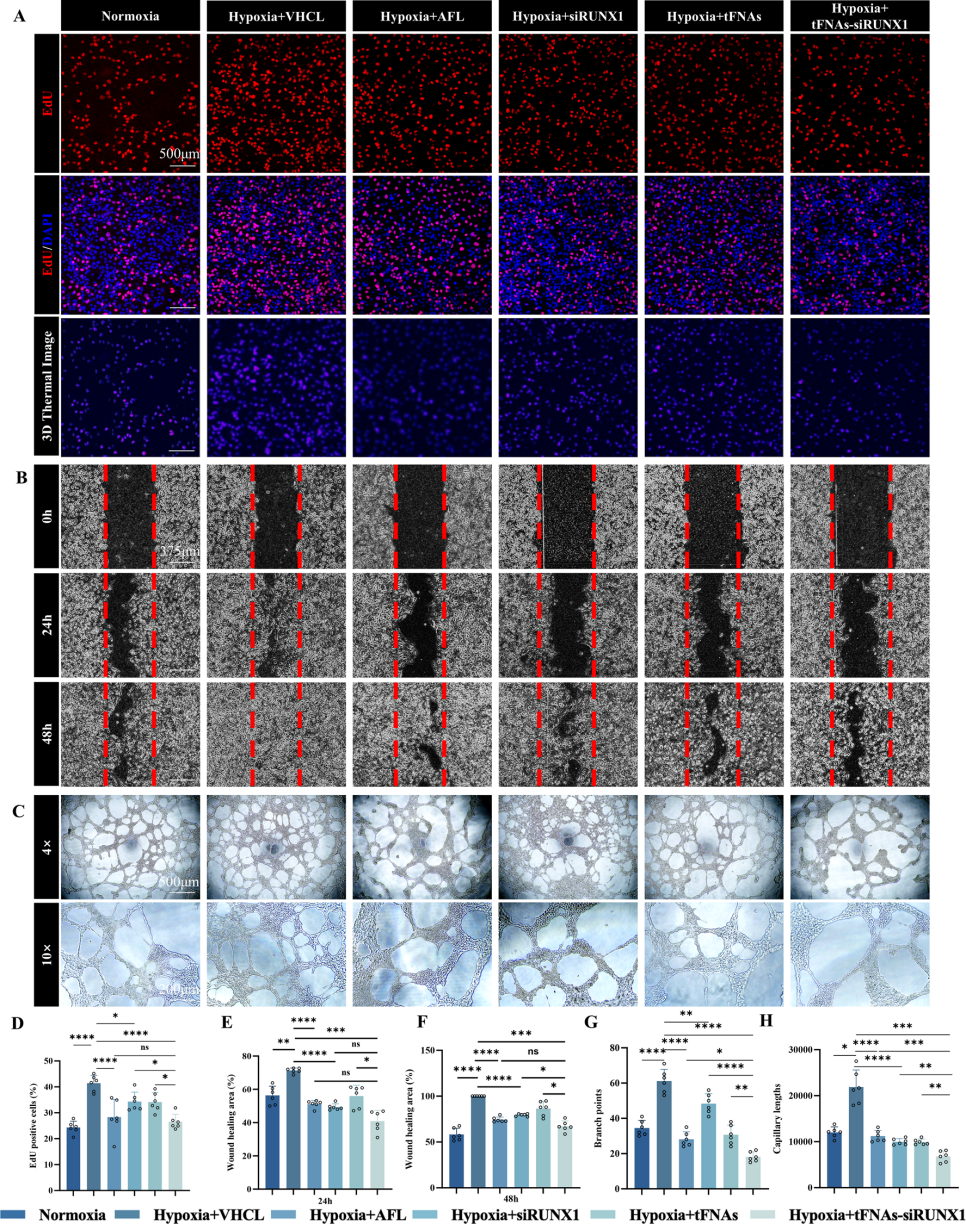
tFNAs-siRUNX1 Inhibit Angiogenesis in vitro. (**A**) Cell proliferation assay results for endothelial cells after different treatments. (**B**) Binary images from scratch-wound assay at 0, 24, and 48 hours after different treatments. (**C**) Tube formation assay results for endothelial cells at 12 hours posttreatment. (**D**) Quantification of Hoechst-positive (*blue*) nuclei colocalized with EdU (*red*). (**E**, **F**) Wound-healing area rates at 24 and 48 hours. (**G**, **H**) Analysis of branch points and capillary lengths. Data: mean ± SD (*n* = 6). Statistical analysis: *P < 0.05, **P < 0.01, ***P < 0.001, ****P < 0.0001 (analysis of variance). *Scale bars*: 500 µm (**A**), 375 µm (**B**), and 500 µm, 200 µm (**C**).

In vitro, to confirm the effect of tFNAs-siRUNX1, HUVECs were segregated into six groups: blank control under normoxia, VHCL control, positive control AFL, Lipofectamine-loaded siRUNX1, tFNAs, and tFNAs-siRUNX1 all under hypoxia. Hypoxia sucessfully induced cell proliferation via the EdU assay, tube formation at both 24 and 48 hours via cell migration ability, scratch-wound assay, and matrigel tubation assay. Lipofectamine-loaded siRUNX1 and AFL served as positive controls. Cell proliferation migration and tube formation were significantly inhibited in both groups if compared with the VHCL (all *P* values less than 0.05). Furthermore, hypoxia-induced cell proliferation, migration ability, and tube formation (Figs. 2A–C) in HUVECs were significantly more suppressed by tFNAs-siRUNX1 treatment compared to VHCL, siRUNX1, or tFNAs alone (Figs. 2D–H). This suppression was similar to that observed with AFL treatment.

### tFNAs-siRUNX1 Reduces RNV and Restores Retinal Vascular Supply in OIR

**Figure 3.**
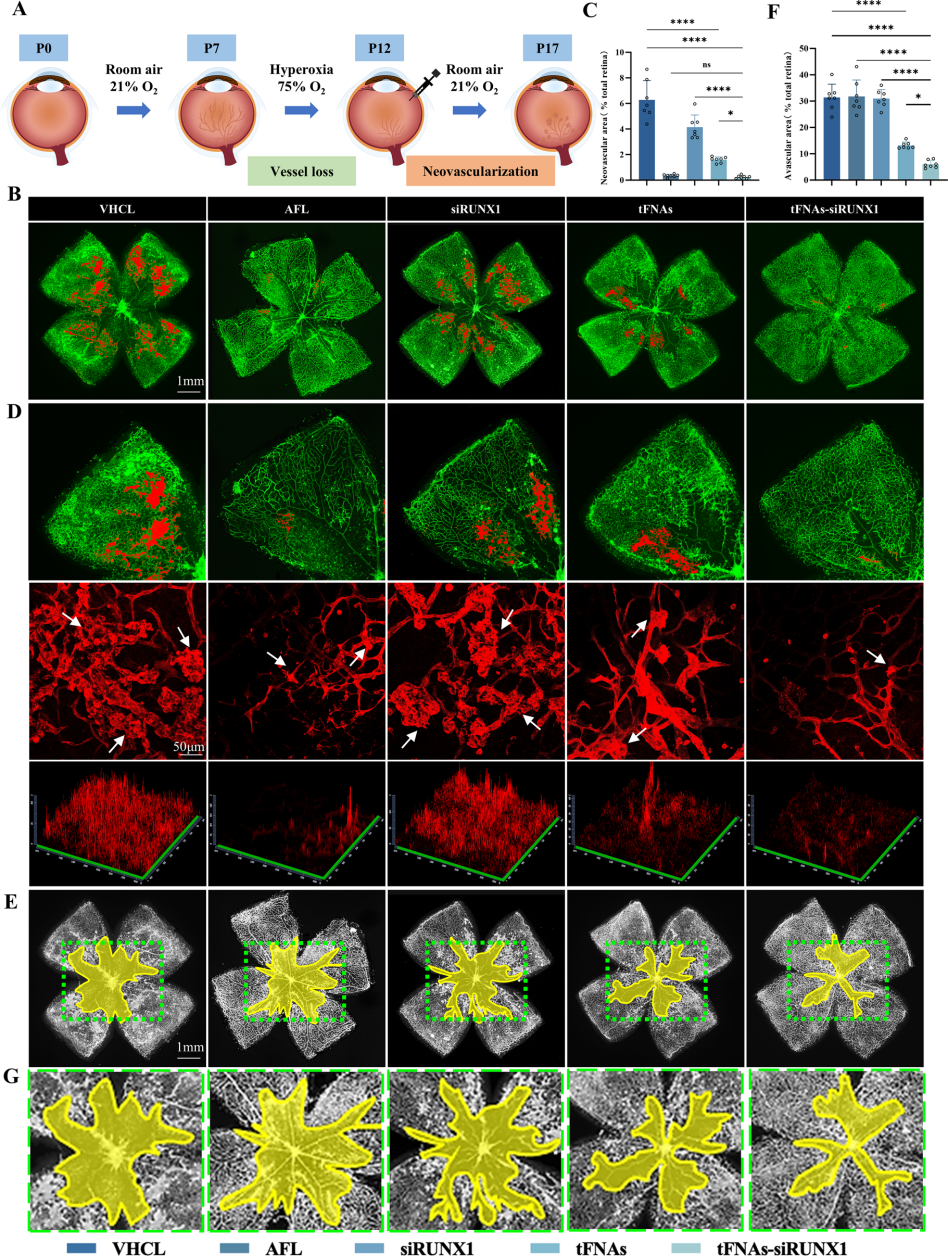
tFNAs-siRUNX1 inhibits retinal neovascularization, reduces the ischemic nonperfusion area, and restores the retinal vessel supply. (**A**) Investigation of the retinal anatomy in vivo using the C57BL/6J mice OIR model. P, postnatal day. (**B**) Representative images of the retinal flat whole mounts stained with IB4 (*n* = 7). (**C**) Analysis of neovascular area (*n* = 7). (**D**) Representative images of neovascular area. The magnified neovascular sprouts (*white arrow*) and 2.5D images from different treatment groups are displayed below. (**E**) Representative images of the retinal whole mounts stained with IB4 showing the NPA outlined in *yellow* in OIR. Magnified images of NPA are displayed below. (**F**) Analysis of avascular area (*n* = 7). (**G**) Magnified images of NPA (*green dotted line*). Statistical analysis: *P < 0.05, **P < 0.01, ***P < 0.001, and ****P < 0.0001 (from analysis of variance test). *Scale bars* are indicated in the figure.

In our study, we administered intravitreal siRUNX1 without any transfection reagent as control,^20^^,^^21^ since there are currently no transfection reagents available for intravitreal use. In the study treating OIR, the results showed that tFNAs-siRUNX1 (0.25% ± 0.12%) decreased RNV area significantly more than that in vehicle control (6.28% ± 1.51%, *P <* 0.0001), siRUNX1 alone (4.15% ± 0.95%, *P <* 0.0001), and naked tFNAs (1.59% ± 0.22%, *P* = 0.014) while being comparable with the AFL group (0.35% ± 0.12%, *P* = 0.9979) (Figs. 3A–C). More importantly, the treatment with tFNAs-siRUNX1 resulted in a significant reduction in the nonperfusion area (NPA) (13.19% ± 1.30%) compared to VHCL (31.41% ± 5.02%, *P* < 0.0001). The RNV area and NPA were significantly decreased in the naked tFNAs group if compared with VHCL (all *P <* 0.0001). Notably, no decrease was observed in the AFL group (31.75% ± 6.27%, *P* = 0.9998) or siRUNX1 group (30.98% ± 3.40%, *P* = 0.9962) if compared to the control (Figs. 3E–G).

### Intraocular Administration of tFNAs-siRUNX1 Effectively Reduces Leakage and Size of Neovascularization

**Figure 4.**
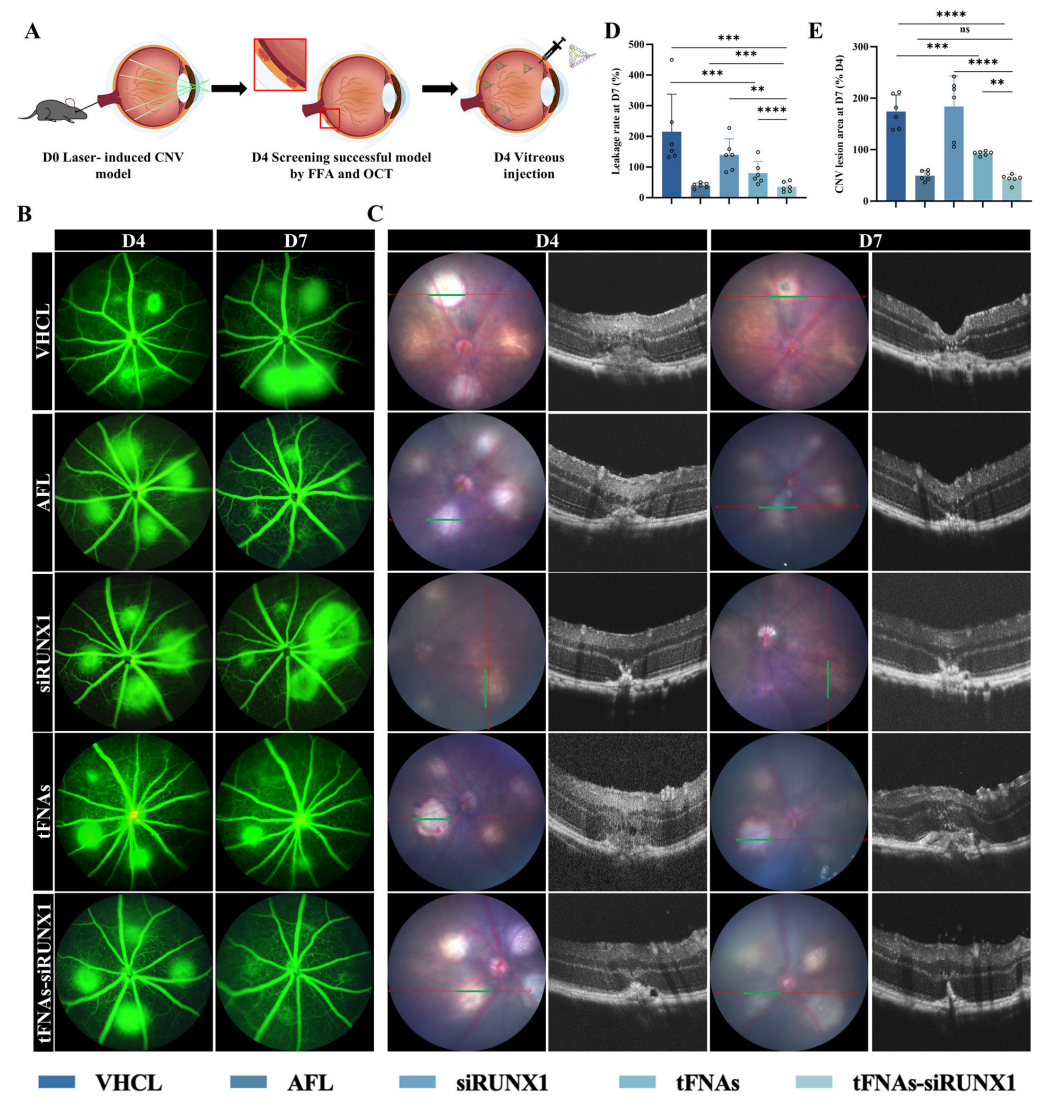
Intravitreal administration of tFNAs-siRUNX1 reduces the leakage of neovascularization. (**A**) A brief description of the experimental procedure conducted in vivo. (**B**) FFA confirmed the success of NV development 4 days (D4) after laser. Seventy-two hours after intravitreous treatment (D7), vascular leakage is significantly reduced in the tFNAs-siRUNX1 group, which is comparable with that in the AFL treatment group and reduced more if compared to the siRUNX1 and tFNAs groups. (**C**) Representative images of funduscopy and OCT (*green line* on the fundus) at D4 and D7. (**D**) Seventy-two hours after tFNAs-siRUNX1 intrvitreous treatment, neovascular leakage area (*n* = 6) and neovascular lesion area (*n* = 6) reduced significantly.

To further assess the therapeutic potential of tFNAs-siRUNX1 for treating vascular leakage from neovascularization (NV), we used a laser-induced neovascularization model in adult mice. The experimental procedure is summarized in Figure 4A. In our study, CNV lesions were identified by their IB4 signal and fluorescence intensity. Briefly, 4 days after laser (D4), the success of the NV model in mice was confirmed using FFA and OCT. For angiography, we administered fluorescein sodium into the mice's blood vessels. The leakage from the new, fragile vessels created a distinct bright circular pattern, which indicated the severity of the NV lesions (Fig. 4B). The lesion area were assessed using OCT (Fig. 4C).

Subsequently, these mice received intravitreal treatments, with follow-up imaging conducted after 72 hours (D7). The results indicated that both the leakage area (24.98 ± 15.31) and lesion area (42.59 ± 8.02) witnessed a significant reduction in the tFNAs-siRUNX1 group compared with those in the VHCL group (197.2 ± 90.82 and 177.60 ± 33.61, *P* < 0.0001, Figs. 4D, 4E) and the tFNAs group (88.62 ± 27.5 and 91.88 ± 4.16, *P* = 0.016 and *P* = 0.020, Figs. 4D, 4E). The leakage area (88.62 ± 27.5) and lesion area (91.88 ± 4.16) were significantly decreased in the naked tFNAs group compared with VHCL (all *P <* 0.0001). No significant differences were found in the siRUNX1-alone group (142.10 ± 47.21 and 172.80 ± 56.76) compared with the VHCL group (*P* = 0.0512, Figs. 4D, 4E).

### tFNAs-siRUNX1 Inhibits Endothelial Cell Apoptosis Through the Bcl/Bax Pathway

**Figure 5.**
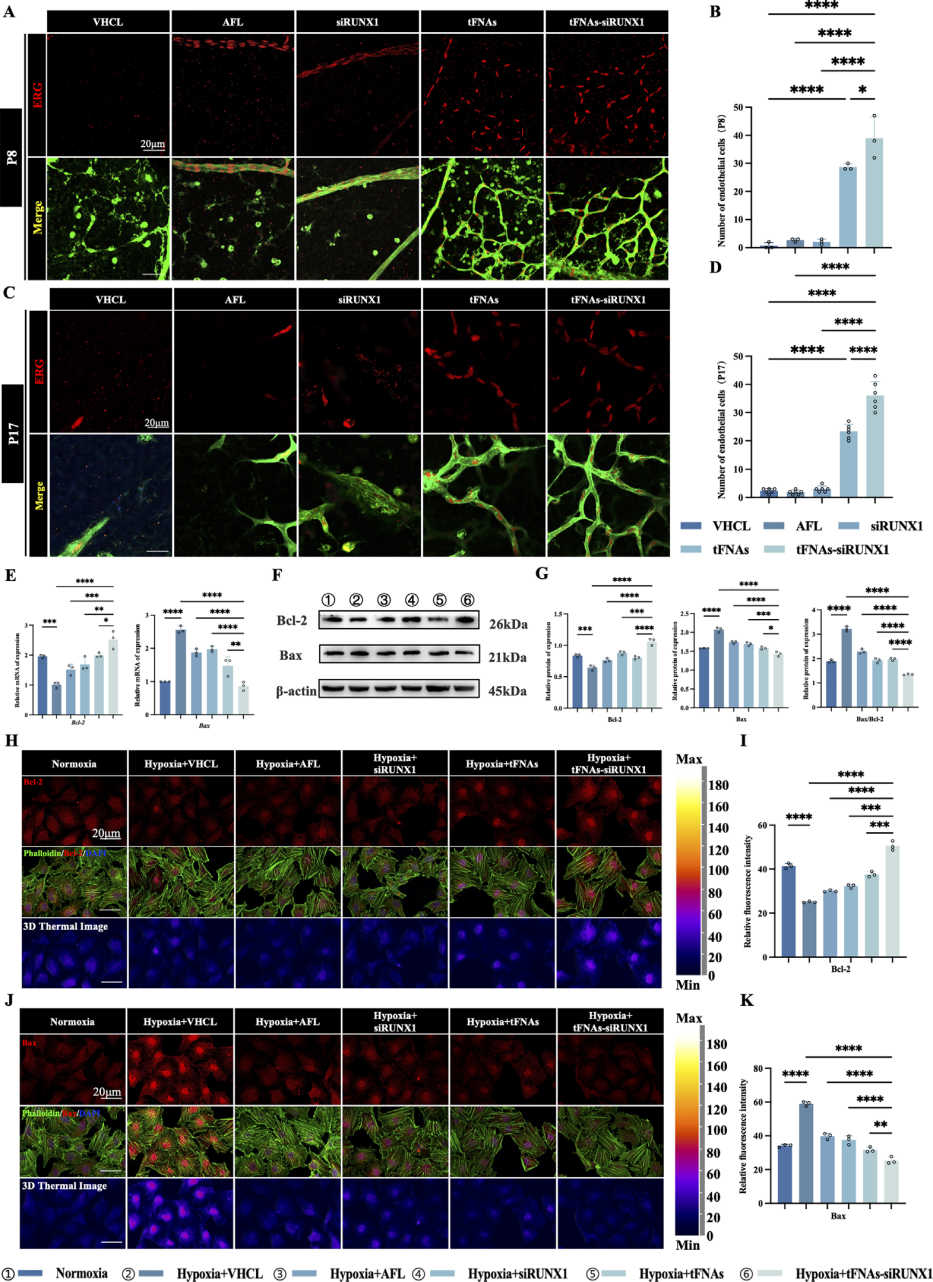
tFNAs-siRUNX1 reverses ECs apoptosis through the Bcl/Bax pathway. (**A**) ECs were double-labeled with IB4 (*green*) and ERG (*red*). At P8, surviving ECs were identified by ERG (ERG^+^IB4^+^). (**B**) The number of ECs in the OIR NPA was significantly higher in the tFNAs-siRUNX1 group compared to other groups (*P* < 0.05). (**C**) ECs double-labeled with IB4 (*green*) and ERG (*red*) on P17 in remodeling vascular networks in NPA. (**D**) The number of ECs in NPA increased significantly after tFNAs-siRUNX1 treatment (*P* < 0.0001). (**E**) RT-PCR revealed decreased *Bcl-2* and increased *Bax* under hypoxia in vitro. In the tFNAs-siRUNX1 group, *Bax* was significantly downregulated and *Bcl-2* was upregulated. (**F**) Western blot analysis confirmed the expression of Bcl-2 and Bax under hypoxia and after treatment. (**G**) Further analysis of protein expression intensity confirmed the downregulation of Bax and upregulation of Bcl-2 after tFNAs-siRUNX1 treatment. (**H**) Immunofluorescence showed lower Bcl-2 (*red*) expression under hypoxia compared to normoxia, with a significant increase after tFNAs-siRUNX1 treatment; phalloidin-labeled cytoskeleton (*green*) is also shown. (**I**) Comparison of Bcl-2 expression levels among different groups. (**J**) In contrast, Bax (*red*) expression was higher under hypoxia compared to normoxia, with a significant decrease after tFNAs-siRUNX1 treatment. (**K**) Comparison of Bax expression levels among different groups. *Scale bars*: 20 µm (**A**, **C**, **H**, **J**).

The occurrence of ischemic retinopathy is mainly related to the damage and apoptosis of endothelial cells (ECs). To assess changes in the number of ECs during regression and reconstitution, we measured the EC counts on P8 and P17 in OIR. ETS-related gene (ERG), a transcription factor linked to angiogenesis, serves as a reliable marker for surviving ECs.^22^ The number of ECs was significantly higher in the tFNAs-siRUNX1 group on both P8 (36.00  ± 4.94, Fig. 5A) and P17 (39.00 ± 7.55, Fig. 5C) compared to other groups (all *P*<0.05). In contrast, the vehicle, AFL, and siRUNX1 groups showed a substantial reduction in EC number, with only a few remaining aside from the main trunk of the retinal vessels (Figs. 5A–D).

We investigated some critical apoptosis-related proteins from the perspective of protein expression in vitro to further investigate the effect of tFNAs-siRUNX1 on the apoptosis of ECs. The Bcl-2 family is the main apoptosis-related proteome, and both apoptotic protein Bax and antiapoptotic protein Bcl-2 belong to this proteome.^23^ In our in vitro study on HUVECs, the *Bcl-2* decreased under hypoxia, while *Bax* and the *Bax/Bcl-2* ratio increased conspicuously. However, in the tFNAs-siRUNX1 group, *Bcl-2* was significantly increased, and *Bax* and the *Bax/Bcl-2* ratio were notably lower compared to other groups (*P* < 0.05, Fig. 5E). The protein expression level of Bcl-2 decreased, while Bax and the Bax/Bcl-2 ratio increased under hypoxia. However, after tFNAs-siRUNX1 treatment, an increase in Bcl-2 and a decrease in Bax and the Bax/Bcl-2 ratio were observed by Western blot and immunofluorescence (all *P* < 0.05, Figs. 5F–K). These findings suggest that tFNAs-siRUNX1 effectively suppresses EC apoptosis under hypoxia or in ischemic nonperfusion retinas.

### tFNAs-siRUNX1 Inhibits RNV and Alleviates Retinal Ischemia Via Regulating the Dll4/Notch1 Pathway

In our study, we used dual staining with IB4 and collagen IV on retinal flat mounts to assess capillary regression. Collagen IV, a key component of the basal lamina, marks regressed vessels. Vessels positive for collagen IV but lacking IB4 immunoreactivity (collagen IV^+^IB4^−^) indicate regression, shown by empty basement membrane sleeves from previously existing vessels.

**Figure 6.**
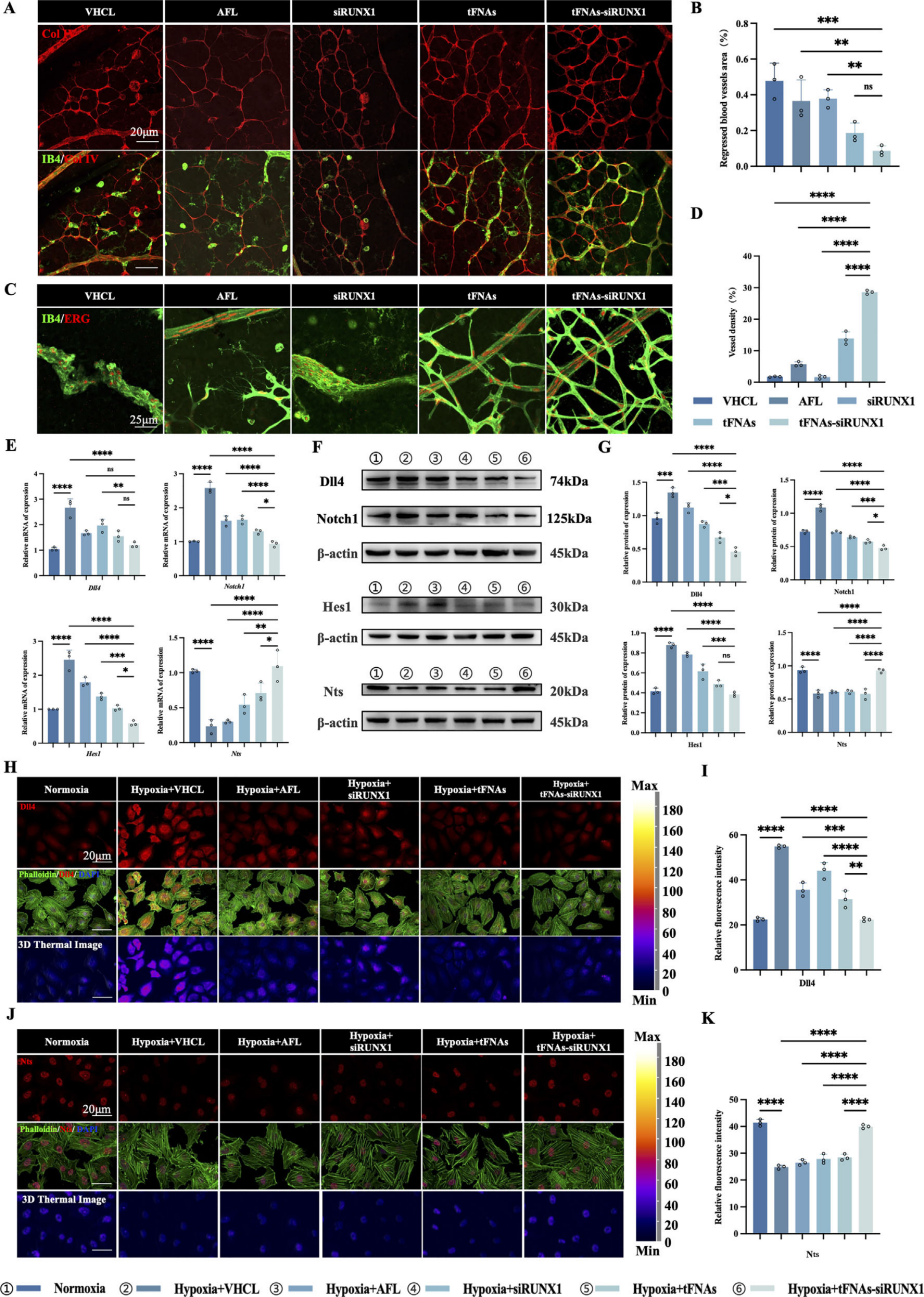
tFNAs-siRUNX1 reverses pathological ischemic retinal responses by regulating the Dll4/Notch1 pathway. (**A**) Blood vessels were double-labeled with IB4 (endothelial cells, *green*) and collagen IV (basal lamina, *red*). At P8, regressed capillary segments were identified by empty collagen IV–positive tubes (collagen IV^+^IB4^−^). (**B**) The percentage of regressed vessels (collagen IV^+^IB4^−^) was significantly reduced after 24-hour treatment with tFNAs-siRUNX1 compared to the siRUNX1 group (*P* < 0.01) and the vehicle group (*P* < 0.001). (**C**) Interconnected vascular networks in the former nonperfusion areas, double-labeled with IB4 (*green*) and ERG (*red*) on P17. *Scale bars*: 25 µm. (**D**) Interconnected vessel density in NPA increased significantly after tFNAs-siRUNX1 treatment (*P* < 0.0001). (**E**) RT-PCR revealed increased *Dll4*, *Notch1*, and *Hes1* and decreased *Nts* under hypoxia conditions in vitro. In the tFNAs-siRUNX1 group, *Dll4*, *Notch1*, and *Hes1* were significantly downregulated, and *Nts* was upregulated. (**F**) Western blot analysis confirmed the expression levels of Dll4, Notch1, Hes1, and Nts under hypoxia and after treatment. (**G**) Further analysis of protein expression intensity confirmed the downregulation of Dll4, Notch1, and Hes1 and upregulation of Nts after tFNAs-siRUNX1 treatment. (**H**) Immunofluorescence showed higher Dll4 (*red*) expression under hypoxia compared to normoxia, with a significant decrease after tFNAs-siRUNX1 treatment; phalloidin-labeled cytoskeleton (*green*) is also shown. (**I**) Comparison of Dll4 expression levels among different groups. (**J**) In contrast, Nts (*red*) expression was lower under hypoxia compared to normoxia, with a significant increase after tFNAs-siRUNX1 treatment. (**K**) Comparison of Nts expression levels among different groups. *Scale bars*: 20 µm (**A**, **H**, **J**) and 25 µm (**C**).

In our study, at P7, pup mice received intravitreal injections of various treatments, and regressed retinal vasculature was assessed 24 hours later on P8 (Fig. 6A). Quantification showed a significant reduction in regressed vessels in the tFNAs-siRUNX1 group (8.60% ± 2.84%) compared to other groups (Fig. 6B), indicating tFNAs-siRUNX1’s potential to prevent acute vascular regression due to elevated oxygen levels. Furthermore, the NPA in the tFNAs-siRUNX1 group decreased by P17. Vessel density significantly increased (28.55% ± 0.62%, *P* < 0.05) compared to other groups (Figs. 6C, 6D), suggesting that tFNAs-siRUNX1 can restore the interconnected vascular network. Adequate blood flow is essential for vessel survival in healthy tissues, whereas stagnant flow due to vessel obstruction leads to rapid regression. Vasoactive proteins, therefore, have a dual role in promoting and inhibiting blood vessel remodeling and regression.^24^ Dll4/Notch signaling regulates multiple genes encoding vasoactive proteins, both vasoconstriction and vasodilation, crucial for retinal angiogenesis.^25^ For the purpose of further exploring the underlying working mechanism, we investigated the Dll4/Notch1 signaling pathway. We hypothesized that the potential protective effect of tFNAs-siRUNX1 in ischemic retinopathy arises from Dll4 inhibition and its associated signaling pathway. Our study observed a significant reduction in Dll4, Notch1, and Hes1 expression and an increase in Nts at both mRNA and protein levels following tFNAs-siRUNX1 treatment in vitro (Figs. 6E–K).

## Discussion

RNV is a primary cause of vision impairment and blindness around the world, which is often triggered by retinal hypoxia from loss of capillary perfusion and endothelial cell depletion, posing a significant threat to visual loss and blindness. Anti-VEGF therapy, the current first-line treatment for RNV, has significant potential drawbacks. RUNX1 has been identified as a key regulator in various neovascular diseases, with its abnormal expression observed in critical cellular populations involved in neovasculization.^8^^–^^10^ This suggests that inhibiting RUNX1 could be a promising new treatment for RNV.^26^ However, due to the poor solubility and limited pharmaceutical potential of commercially available RUNX1 inhibitors,^11^^,^^12^ an effective and efficient delivery system is needed. In our study, we explored the treatment effect and the molecular mechanism of tFNAs-siRUNX1, an innovative DNA nanocomplex, in ischemic retinal RNV.

Interestingly, although tFNAs are considered “naked,” they still exhibit some antiproliferative and antitubulation effects, as observed in our study and others.^16^^,^^27^ Previous reports have demonstrated that naked tFNAs, despite lacking additional targeting moieties, can exert notable effects on cell proliferation and tube formation in certain cell types.^28^^–^^31^ More importantly, when siRUNX1 is delivered via tFNAs, the synergistic effect significantly enhances the therapeutic efficacy, amplifying the suppression of RNV.

We confirmed that tFNAs-siRUNX1 were successfully synthesized and penetrated by cells using different methods, which laid the foundation for our subsequent experiments. Inconsistent with previous studies, DNA nanomaterials exhibited excellent stability and biological applications.^16^^–^^18^ To be different from before, we purified the compounds, following the methods described in our previous research.^16^^–^^18^ This process allowed us to remove excess bases, mismatched sequences, and stray single strands, so we ended up using a lower concentration, 100 nM tFNAs-siRUNX1, for in vitro and in vivo experiments than the previous study.^17^ Hypoxia is a well-known form of stress that impairs the biological function of cells.^32^ The oxidative stress and generation of reactive oxygen species (ROS) may induce RUNX1 expression in HUVECs.^33^ These factors activate quiescent ECs and promote cell proliferation, migration, and vascular permeability. Several cellular functional assays, including the EdU cell proliferation assay, tube formation assay, and wound-healing assay, were conducted, and the results showed that tFNAs-siRUNX1 significantly decreased proliferation, migration, and tube formation of HUVECs.

It is well documented that current anti-VEGF agents, including AFL, have no effect on alleviating the ischemic nonperfusion area.^34^ Our results further confirmed that AFL showed no improvement in nonvascularized regions or hindered the reestablishment of blood supply in the ischemic retina in the OIR model.^34^^,^^35^ In contrast, tFNAs-siRUNX1 effectively inhibited retinal ischemia and facilitated restoration of the retinal vessel supply. Our current research has highlighted tFNAs as a promising tool in biology due to their exceptional properties. It showed that tFNAs-siRUNX1 inhibit RNV effectively, even better than the naked tFNAs. The combination of tFNAs with siRUNX1 could have the potential to significantly enhance efficacy, comparable to the AFL. More excitingly, in our current tFNA-siRUNX1 nanopaticles, it looks like it addresses ischemia and restores the retinal blood supply, with the restored vessel supply sharing the normal structure and perfusion.

The OIR model, which is conducted in newborn mice with very small eyes, is not suitable for evaluating neovascularization leakage. To determine the therapeutic potential of tFNAs-siRUNX1 for treating vascular leakage from NV, we used a laser-induced neovascularization model in mice. Laser photocoagulation selectively destroys the retinal pigment epithelium, Bruch's membrane, and choroid capillaries, triggering an imbalance of angiogenic factors and leading to new blood vessel formation. These new vessels lack connective tissue, a basement membrane, and receptors for blood flow, making them fragile and prone to rupture and leakage. A previous study proved that naked tFNAs could regulate the inflammatory process of CNV by polarizing macrophages, thereby improving the symptoms of CNV.^17^ Notably, our experiments demonstrated that tFNAs with siRUNX1 effectively reduce fundus leakage. These findings suggest that tFNAs-siRUNX1 could emerge as a powerful, novel DNA-based pharmaceutical candidate for managing neovascularization. The potential mechanism should be explored in future research.

Our previous study found that naked tFNAs could mainly regulate the PI3K/AKT/mTOR signaling pathway to inhibit endothelial cell growth and metabolism under hypoxia, thus inhibiting pathological neovascularization and suppressing NPA in the ischemic retina.^16^ The better effectiveness of tFNAs-siRUNX1, in both inhibiting the NV and restoring the retinal vessel supply, was identified in our study. It was reported by Grant et al.^23^ that blockade of endothelial apoptosis, preserving ECs within ischemic tissue, could promote revascularization in ischemic tissue. According to this, we investigated EC apoptosis using the OIR model, in which vessel closure and EC apoptosis caused capillary regression, retinal ischemia, and eventually neovascularization.^36^ Apoptosis is governed by two primary pathways: one involving Bcl-2 family proteins and the other involving cell surface “death receptors.” Notably, research has highlighted the significant role of Bcl-2 family proteins in regulating EC apoptosis.^23^ This family is divided into prosurvival and proapoptotic groups, and their interaction determines the activation of apoptosis effector proteins Bcl-2 and Bax.^37^^,^^38^ Our findings suggest that tFNAs-siRUNX1 suppresses EC apoptosis under hypoxia or in ischemic nonperfusion retinas by regulating the Bcl-2/Bax pathway more effectively than naked tFNAs.

It was demonstrated that EC clusters, which were protected against apoptosis, actively contributed to the reestablishment of a functional vascular network during revascularization of the ischemic retina. However, inhibiting apoptosis in ECs does not effectively impede vessel regression induced by exposure to elevated levels of oxygen.^25^ These findings indicate that the immediate factor leading to vascular regression in OIR is likely attributed to capillary nonperfusion, and EC apoptosis in OIR has previously been proposed to occur secondary to loss of blood flow.^25^^,^^39^ The inhibition of Dll4/Notch was confirmed to enhance blood vessel sprouting, resulting in an accelerated revascularization process in the ischemic areas of the retina within the OIR model.^40^ The expression of multiple genes in the retinal vasculature following angiogenesis was found to be differentially regulated by Dll4/Notch signaling. These genes encode vasoactive proteins that play a crucial role in regulating vasoconstriction and vasodilation.^24^ Our findings suggest the potential protective effect of tFNAs-siRUNX1 in ischemic retinopathy arises from Dll4 inhibition and its associated signaling pathway, which regulates the expression of the vasoactive gene neurotensin (Nts).

Overall, our study suggested that tFNAs-siRUNX1 can mitigate Bcl-2/Bax-mediated apoptosis, leading to a partial reduction in initial perfusion loss or vascular breakdown that ultimately results in retinal ischemia. The revascularization effect of tFNAs-siRUNX1 enhances the survival and persistence of endothelial cells during vessel regression, preventing their usual demise. These endothelial cells form fragmented clusters within ischemic regions, leading to less vessel regression and ultimately reestablishing a new connected vascular network. Dll4/Notch signaling, regulating the expression of the vasoactive gene Nts, influences vessel closure in the OIR model.^25^

## Conclusions

**Figure 7.**
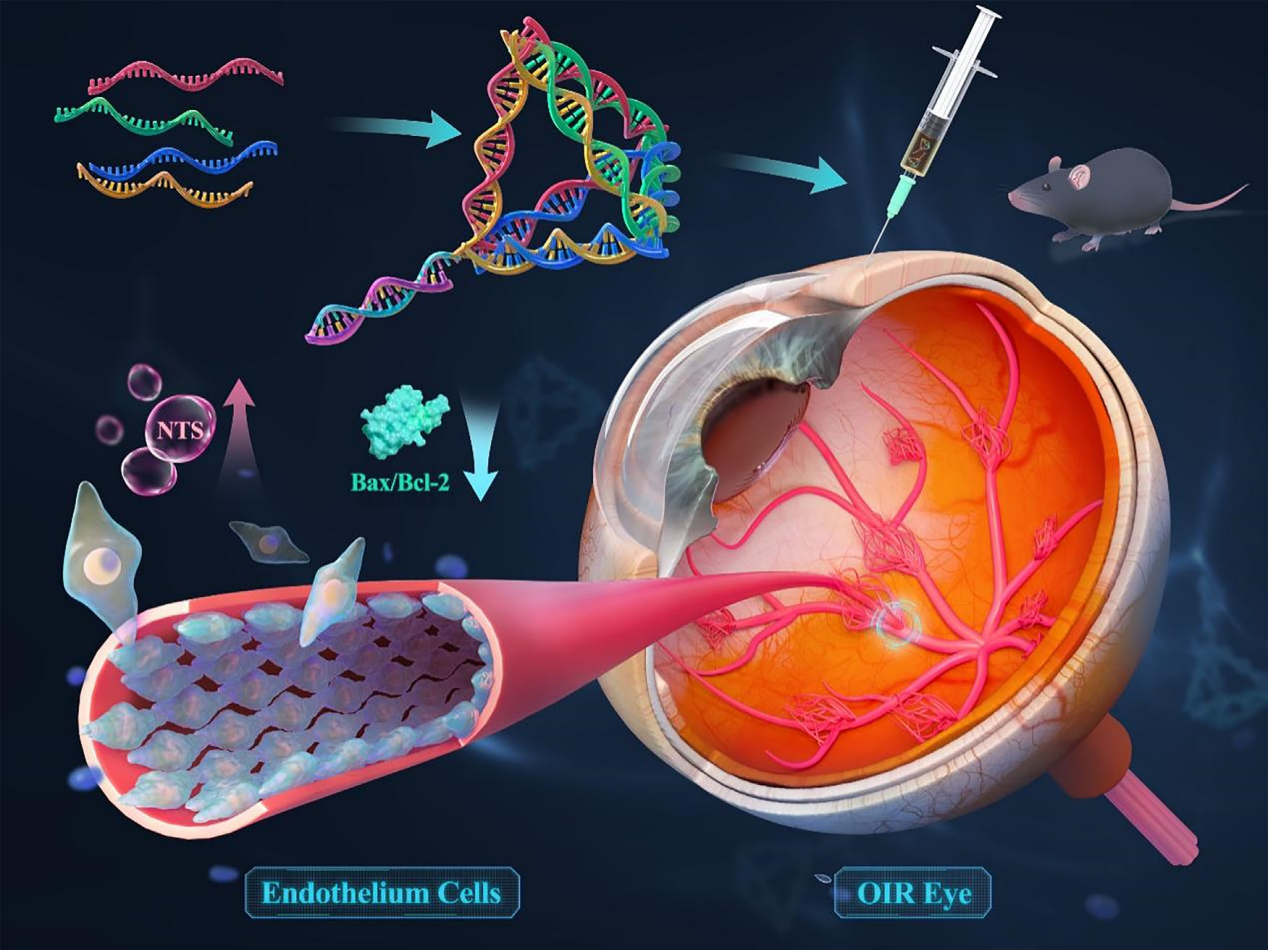
Tetrahedral framework nucleic acid system mitigates EC loss, forestalls vascular regression, and maintains EC clusters via Dll4/Notch1 pathways in the ischemic condition.

In summary, we developed a straightforward and effective approach for delivering RUNX1 siRNA embedded in tFNAs and utilizing them for in vitro and in vivo models. The findings demonstrated that tFNAs efficiently and safely delivered siRUNX1 to target endothelial cells in hypoxic and ischemic retinas. The synergistic effect of tFNAs and siRNUX1 was observed to effectively diminish neovascularization, alleviate retinal ischemia, and restore the retinal blood supply, demonstrating tFNAs-siRUNX1’s potential in addressing retinal neovascularization disorders (Fig. 7). More importantly, our study unveiled that tFNAs-siRUNX1 effectively suppressed Dll4 and modulated the Dll4/Notch1 signaling pathway, leading to augmented activation of Nts and consequent prevention of EC apoptosis. This approach demonstrates significant potential as a crucial treatment for retinal neovascularization and may offer a viable solution for its cure, holding substantial promise for future clinical applications.
